# New Insights into the Generation of CD4 Memory May Shape Future Vaccine Strategies for Influenza

**DOI:** 10.3389/fimmu.2016.00136

**Published:** 2016-04-11

**Authors:** Priyadharshini Devarajan, Bianca Bautista, Allen M. Vong, Karl Kai McKinstry, Tara M. Strutt, Susan L. Swain

**Affiliations:** ^1^Department of Pathology, University of Massachusetts Medical School, Worcester, MA, USA

**Keywords:** influenza, vaccination, memory checkpoint, late-antigen, CD4 T cells, cell-mediated immunity

## Abstract

Influenza viral evolution presents a formidable challenge to vaccination due to the virus’ ability to rapidly mutate to evade immune responses. Live influenza infections generate large and diverse CD4 effector T cell responses that yield highly protective, long-lasting CD4 T cell memory that can target conserved viral epitopes. We review advances in our understanding of mechanisms involved in generating CD4 T cell responses against the influenza A virus (IAV), focusing on specialized follicular helper (T_FH_) and CD4 cytotoxic (ThCTL) effector subsets and on CD4 T cell memory. We also discuss two recent findings in context of enhancing vaccine responses. First, helper T cells require priming with APC secreting high levels of IL-6. Second, the transition of IAV-generated effectors to memory depends on IL-2, costimulation and antigen signals, just before effectors reach peak numbers, defined as the “memory checkpoint.” The need for these signals during the checkpoint could explain why many current influenza vaccines are poorly effective and elicit poor cellular immunity. We suggest that CD4 memory generation can be enhanced by re-vaccinating at this time. Our best hope lies in a universal vaccine that will not need to be formulated yearly against seasonal antigenically novel influenza strains and will also be protective against a pandemic strain. We suggest a vaccine approach that elicits a powerful T cell response, by initially inducing high levels of APC activation and later providing antigen at the memory checkpoint, may take us a step closer to such a universal influenza vaccine.

## Introduction

Efforts to develop a vaccine for influenza date back to 1936 when the first live attenuated virus vaccine was produced in chicken eggs (1). Eighty years later, an effective influenza vaccine remains elusive, with the CDC reporting an overall vaccine efficacy of only 23% in the 2014–2015 influenza season (2). While influenza pandemics occur rarely, the H1N1 pandemic in 2009 also reminded us that they remain a major threat (3, 4). In this review, we discuss how CD4 T cells combat influenza viruses, with a focus on CD4 memory generation. We suggest strategies for improved influenza vaccines based on our new understanding of the mechanisms by which CD4 memory and functionally specialized effectors are generated.

## Influenza Viruses and the Immune System – A Constantly Evolving Challenge

Influenza A (IAV) and influenza B viruses infect humans causing widespread, sometimes fatal, disease. Both viruses contain eight gene segments, which encode surface proteins involved in viral attachment, entry and release from the cell, and internal proteins that predominantly play a role in viral replication (5–7). The two coat proteins, hemagglutinin (HA) and neuraminidase (NA), on the outer envelope are used to subtype the virus.

Each year new variants of influenza viral strains become dominant because of the high rate of mutations of the RNA virus. “Antigenic drift,” one of the strongest drivers of viral evolution, results from the error-prone replication process (6) in conjunction with immune clearance based primarily on recognition of HA and NA by neutralizing antibody (Ab) (6). By virtue of their surface expression and abundance, the HA and NA contain the dominant epitopes recognized by Ab and to a lesser degree by responding T cells (8). Viruses having mutations in key epitopes can evade the immune system resulting in yearly drift within strains (9–12). “Antigenic shifts” that cause pandemics arise when re-assortment of influenza virus gene segments, often from different host species, occur (13).

Current vaccines rely largely on the induction of influenza-specific Ab (14). New influenza vaccines are needed yearly to target the mutated epitopes in circulating strains predicted to dominate the upcoming flu season, but the predictions can be wrong leading to poor vaccine efficacy like that seen over the past two flu seasons (15–17). Antigenic mutations also make vaccine production logistically difficult as viruses can also mutate during the processes used in vaccine production (18).

Studies of viral evolution over the years indicate that while only 2.7% of epitopes recognized by Ab are conserved, 15% of T cell epitopes remain unchanged (8). This higher conservation of T cell epitopes correlates with the ability of T cells to target internal viral proteins involved in replication, which are far less tolerant to selection pressure compared to the external coat proteins (19–21). For example, the HA and NA of the pandemic H1N1 strain have acquired mutations at a rate six to eight times faster than the internal NP protein, in terms of amino acids substitutions per site per year (22). Ultimately an ideal vaccine that also combats viral escape would be one that elicits a broad immune response against the whole virus – a response which includes CD4 and CD8 memory responses in addition to Ab responses.

Thus, we suggest that a better understanding of the generation of T cell memory could lead to the development of vaccine strategies that induce more memory T cells, which are better able to recognize yearly influenza strain variants and new pandemic strains, providing longer-lived protection.

## CD4 Effector Responses Against Influenza

The first line of immune defense upon infection is comprised of PRR (pattern recognition receptor) pathways induced by the virus in infected epithelium, DCs, alveolar macrophages, and other myeloid cells triggered by viral PAMPs (pathogen associated molecular patterns) that act to induce innate defenses upon infection (23). PRR-activated cells also produce the inflammatory cytokines that promote the APC activation required for optimal T cell priming and costimulate adaptive T and B cell responses (23).

Activated APC migrate to the secondary lymphoid organs as early as 2 days post infection to present antigen to naive T cells (24). Effective CD4 T cell activation requires three distinct signals: antigen recognition by TCR, costimulation of CD28 on the T cell by CD80/86 on APC, and APC-produced costimulatory cytokine(s). Together, these signals drive CD4 T cell activation, with the cytokine milieu being a major factor in determining polarization into Th1, Th2, or Th17 subsets (25, 26). Regulatory T (T_REG_) cells are also induced during influenza infections (27–29).

Influenza A virus infection predominantly induces a Th1 response, with most CD4 effectors producing IFNγ though the importance of IFNγ in combating the flu has been debated (26). Th17 effectors that contribute to protection are produced during IAV infections (30), but they also contribute to immunopathology (31). The polarization of T helper subsets during IAV infections has been previously reviewed (25, 26) and will not be discussed further.

While one of the classical functions of CD4 T cells is to help CD8 T cell effector generation, such help is not important for an effective primary immune response to influenza (32). However, recent studies have shown an important role for CD4 help during CD8 priming in the formation of CD8 resident memory T (T_RM_) cells in lung airways during influenza infection (32, 33). CD4 T cells also regulate CD8 effector responses during IAV infections by modulating IL-10 production by CD8 T cells (34) and by counteracting T_REG_ suppression (35).

Two other functionally specialized CD4 effector subsets that appear later in the response are T follicular helper cells (T_FH_) and cytotoxic CD4 T cells (ThCTL). T_FH_ promote T-dependent B cell responses. T_FH_ lineage fate is determined in part by expression of transcription factor Bcl6 followed by upregulation of CXCR5, which causes the helper T cell to migrate to the germinal center (36). T_FH_ drive B cell survival, proliferation, class switching, plasma cell differentiation, and somatic hypermutation that take place in germinal centers (37). Germinal center T-dependent B cell responses are also required for generating memory B cells and later long-lived plasma cell (LLPC)-derived Ab responses that confer protection against reinfection (25, 38, 39). Direct evidence of the importance of the T_FH_ subset in mounting an immune response against IAV comes from human influenza vaccine studies that correlate efficacy of protective Ab responses generated to the number of T_FH_ cells detected in the blood (40–43).

Cytotoxic CD4 T cells are effector CD4 T cells that mediate perforin-dependent, MHC Class-II specific cytotoxic activity. They are tissue-restricted, and in mouse models of IAV, infections are seen only in the lung (44–46). Although MHC-II expression is restricted to APC under steady state conditions, MHC-II is upregulated on infected epithelial cells in the lung during IAV infection (45). ThCTL could therefore mediate clearance of infected epithelial cells and contribute to clearance of the virus. Their contribution to the immune response against IAV is, however, often masked by other multiple redundant effector mechanisms (44, 47). The regulation and differentiation of this subset remain to be fully elucidated, though recent studies in a mouse model of IAV suggest a role of Blimp-1 and type I interferon pathways in ThCTL formation (48). Thus, ThCTL represent a uniquely regulated CD4 T cell subset to target in vaccine approaches.

## Aging Impairs CD4 T Cell Helper Responses Against Influenza

Influenza infections cause extensive morbidity and mortality among the aged and current vaccines fail to provide widespread protection in this at-risk subset of the population (49, 50). B cell Ab responses (49), especially IgG responses (51) and induction of LLPC key to protection against re-infection, are impaired in aged mouse models. We have found that, in mice, reduced naive CD4 T cell responses can be enhanced by activation of the APC by TLR agonists, and this dramatic effect is dependent on IL-6 (52, 53). Young naive cell responses are also enhanced by the same mechanism (50), as are responses of aged human CD8 T cells (49, 54). Thus, we suggest that strategies, which couple antigen with agents that specifically activate relevant APC to produce IL-6 may improve vaccines for the aged.

## CD4 T Cell Memory

Memory CD4 and CD8 T cells can provide strong protection in the absence of neutralizing Ab following heterosubtypic infection in mouse models of IAV infection (45, 47, 55). Memory T cells were originally thought to be stem-like, retaining pluripotent potential (56), but new developments have demonstrated that memory T cells generated following infection are mostly composed of multiple highly differentiated subsets, which mediate the enhanced protective ability of memory over naive T cells.

Memory CD4 T cells generated by live infection retain some of the differentiation-associated changes attained during the effector phase of the response. CXCR5 expressing memory CD4 T cells are capable of enhanced B cell help during re-challenge, although they are also capable of differentiation into many different cytokine-secreting subsets during the secondary response (55, 57, 58). Th1-like memory cells were identified in one report by a lack of CXCR5 expression and in another by increased Ly6C expression. Using either method, isolated memory cells largely became IFNγ-producing Th1 cells during the secondary response, demonstrating far less plasticity than the CXCR5^+^ memory T cells (57, 58). This retention of functional imprinting that occurs in the effector phase may account for some of the enhanced function during the secondary response (59).

Lung CD4 T_RM_ cells have recently been characterized following influenza infection. This subset, which is thought to be present at the frontline of infection in tissues, is critical for protection against a lethal dose of IAV (60). While studies have demonstrated that antigen recognition in the lung along with TGFβ signals are required for the formation of CD8 T_RM_ during influenza infection, it is unclear if CD4 T_RM_ cells have similar requirements (61). Both lung T_RM_ CD4 and CD8 T cells express CD69, but only CD8 T_RM_ cells express CD103 (62), suggesting the two populations may occupy different niches within the lung or that they may be differently regulated.

Memory CD4 T cells provide protection via multiple synergizing mechanisms including both IFNγ-dependent and -independent mechanisms, cytotoxic mechanisms, help for B cells and rapid induction of innate inflammatory responses (25, 26, 47). Additionally, the presence of cross-reactive memory CD4 T cells has been correlated with less severe disease following heterosubtypic infection in humans (63). Therefore, the ability to induce CD4 memory responses is central to the development of vaccines that are more potent, broader in specificity and would also benefit from the synergy of multiple functional pathways to eliminate the virus.

## Late-Antigen Shapes Efficient T Cell Effector and Memory Responses

Viral clearance occurs by days 10–13 of IAV infection, but antigen presentation can occur for up to 3 weeks (64). While early priming events are sufficient for some effector and memory T cell differentiation (65–67), recent studies have identified a role for signals received at the effector stage in shaping ongoing CD4 T cell responses. CD28 signals, after priming, have been shown to be required for full Th1 and T_FH_ differentiation in IAV (68). A second round of antigen recognition is also required for full T_FH_ differentiation (69–71).

Multiple studies have suggested that CD4 T cells require longer periods of antigen stimulation during antigen priming compared to CD8 T cells for effector function and proliferation (72–75). Some studies have also found that CD8 effector T cells require antigen stimulation after initial priming, out to 8 days after IAV infection for continued proliferation and survival (76, 77). One study identified a defined window from days 5 to 8 following administration of initial antigen, when antigen with adjuvant prevents apoptosis of effector CD8 T cells (78). Therefore, while the requirements during priming may be different for CD4 and CD8 T cells, it appears their response to antigen at the effector stage may be similar.

In addition to shaping effector responses, recent studies have highlighted the role of late signals in promoting the formation of functional, protective memory T cell populations. Late-antigen recognition, 5–8 days after infection, promotes the formation of protective memory CD8 T cells (79, 80). In the IAV infection model, we found that costimulation and IL-2 signaling during 5–7 days after initial priming are required for almost all CD4 memory cells to form (81). We have defined this time, 5–7 days after primary antigen encounter, as the “memory checkpoint.” Our study also showed that cognate MHC-II interactions and CD27–CD70 interactions are required at this checkpoint for efficient transition of CD4 effectors to memory (81).

It is well established that during chronic infections, antigen persistence drives T cell exhaustion (82). Some studies show that TCR stimulation 7 days after the response results in apoptosis of CD8 T cells (78). Others have found that after 10 days of antigen stimulation, CD4 T cells begin to express an exhausted phenotype (83). Thus, the memory checkpoint appears to be a tightly regulated time window during which antigen presentation has a substantial impact on the effector and memory T cells generated, beyond which antigen may drive exhaustion.

Some have found that late-antigen presentation to CD8 T cells occurs primarily in the lung (61, 77, 84), while others believe it also occurs in the lung-draining lymph nodes (85). It is yet unclear where antigen presentation during the effector phase occurs for CD4 T cells. It is conceivable that antigen recognition during the CD4 effector phase also occurs in the SLO since T_FH_ must recognize late-antigen to provide help (36). We speculate that antigen recognition at the effector phase in the SLO provides signals that generate a circulating, central memory population, while antigen recognition in the lung during the effector phase may lead to T_RM_ formation.

Thus, although small numbers of less differentiated memory cells may be formed following initial priming alone, additional cognate interactions are required at the “memory checkpoint” during the effector phase for the formation of large, functional CD4 memory populations (81). Given the many ways distinct CD4 subsets orchestrate the immune response, their continued dependence on antigen and costimulation for effector function and further differentiation may dictate the fate of distinct CD4 subsets and thus tailor the response.

## Perspective on Translating Recent Advances into Improved Influenza Vaccines

**Figure 1:**
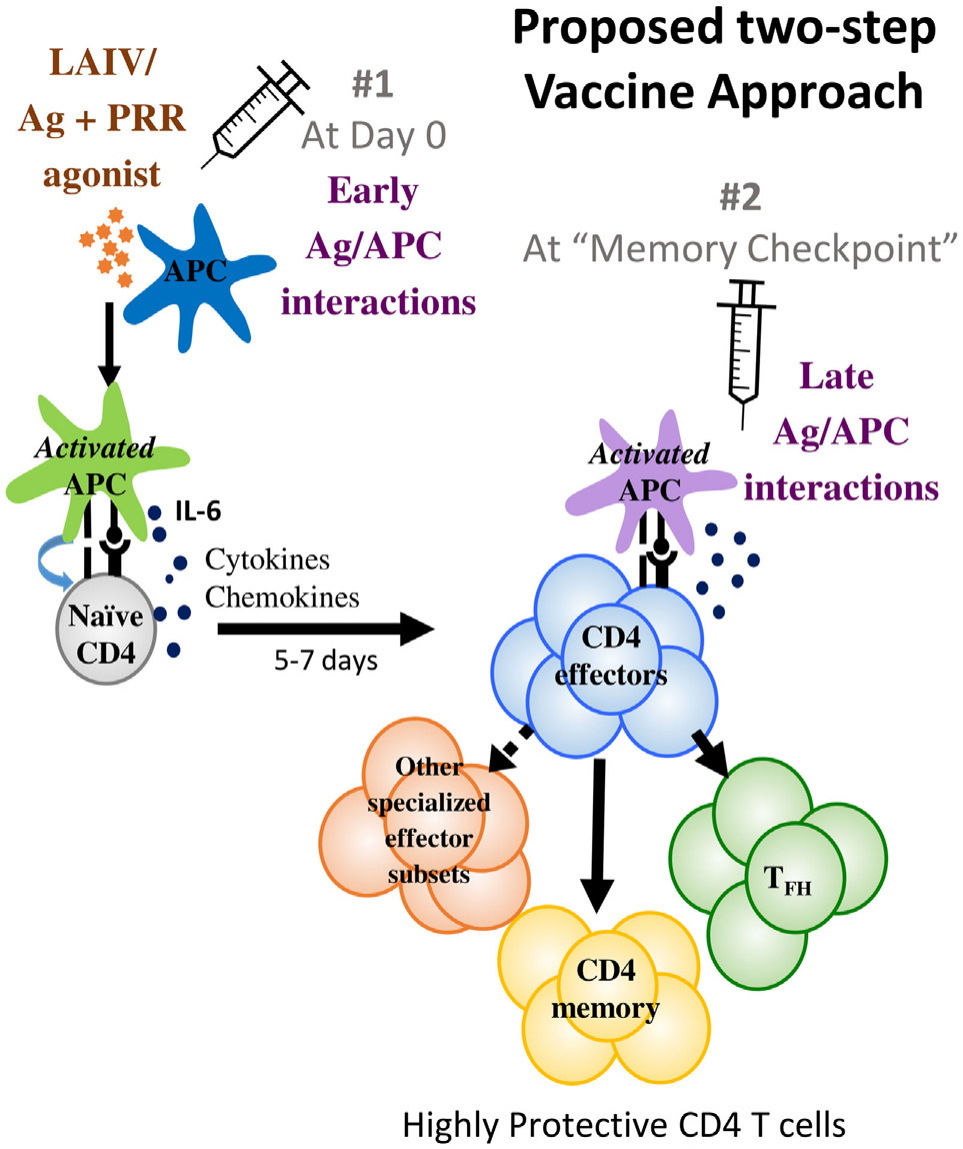
**We propose a two-step vaccine approach that optimizes antigen presentation at two different phases**. The first phase is the early antigen–APC interaction phase that primes T cell responses where APC activation to optimize IL-6 production is suggested. The second phase is the memory checkpoint when antigen on activated APC drives the differentiation of effectors to specialized CD4 subsets such as T_FH_ and perhaps other specialized effector subsets and also induces optimal CD4 memory formation. For IAV infections, we suggest that providing antigen 5–7 days after priming would be a suitable timeframe for administering the second vaccine dose that would provide late-antigen.

Given the challenges posed by influenza viruses and the recent advances in understanding CD4 T cell immune responses and memory summarized above, we suggest new strategies at two different levels that may enable an improved vaccine (Figure 1).

There are currently three classes of seasonal influenza vaccines in use: inactivated influenza vaccines consisting of both split-virion and subunit vaccines (IIV), live attenuated influenza vaccines (LAIV), and recombinant HA vaccines (14, 86). The multivalent vaccines contain components of both type A and type B viruses predicted to circulate in the upcoming influenza season. Both inactivated and new recombinant vaccines (commercially known as FluBlok^®^) consist of HA with or without NA purified from viruses cultured in eggs or in cell culture and inactivated or made as a recombinant protein using baculovirus expression systems in insect cells, respectively (87). Live attenuated vaccines (commercially known as FluMist^®^) are composed of cold-adapted viruses that do not survive at temperatures above 37°C and thus only infect the upper respiratory tract in humans and cause very mild infections sufficient to elicit modest immunity (14).

Both LAIV and IIV have been demonstrated to be equally effective in adults, while in children LAIV has demonstrated superior efficacy in various studies (86). This increased efficacy in children has been attributed to the wider range and longer-lived immune responses that LAIV elicits. While IIV primarily elicits IgG serum Ab responses against HA, LAIV has been shown to elicit a wider range of Ab responses including IgG and IgA against both HA and NA viral proteins. LAIV responses also promote CD4 and CD8 T cell responses against internal viral proteins and if these become memory T cells, they could be cross-protective against antigenically novel pandemic as well as seasonal epidemic influenza strains (8, 88).

Replicating live virus is likely to be the best at inducing APC activation through PRR pathways, a critical step in an effective helper T cell response. Production of IL-6 by the activated APC would also contribute to enhanced priming of young and aged responses, as discussed. This would explain the enhanced efficacy of LAIV compared to IIV. Partially purified IIV may induce minor activation through the remaining RNA and DNA present, while recombinant or highly purified subunit proteins are likely to be devoid of most PRR-inducing ability. Adjuvants could be used to enhance PRR responses in APCs, though further research will be required to achieve optimal activation without causing widespread inflammation (89). DC vaccines targeting antigen to PRR-activated APCs could also potentially be used to achieve similar strong initial T cell responses (90).

However, even with LAIV, it is likely the level of antigen presentation decreases with time since replication does not occur in the lung and is not likely to be high during the effector phase of the T cell response (91, 92), through the memory checkpoint, when such presentation is important for efficient T cell memory induction. Data also suggest that antigen presentation specifically in the lung is important to drive CD8 T_RM_ responses to IAV (61). Thus, we suggest that additional antigen that can be presented by activated APC could be administered at the effector checkpoint through the intranasal route, a hypothesis we are currently testing in the mouse model. A vaccine strategy including an early “boost” has indeed generated superior CD8 memory in mice thus lending support to the concept (93). We suggest that additional antigen priming at the memory checkpoint would both enhance T cell memory and potentially increase T_FH_ responses, which also require late-antigen encounter and are correlated directly with successful Ab titers and viral clearance.

A more robust “T cell” vaccine incorporating these strategies should promote larger and longer lived Ab responses like those achieved by viral infection and generate more multi-functional memory T cells that are cross-reactive to antigenically novel strains. This might give us a recipe for a more “universal” influenza vaccine that would not need to be reformulated every year and would provide some protection against potentially pandemic strains.

## Author Contributions

PD organized the different sections of the manuscript, coordinated other author contributions, put together the sections in the manuscript, sent out for review to all authors, and finalized the manuscript for submission. BB wrote the section on T cell memory and on Late-antigen, helped organize different sections of the manuscript, and helped finalize the manuscript for submission. AV, KM, and TS helped organize different sections of the manuscript, contributed their insights on all sections in the manuscript, and helped finalize the manuscript for submission. SS wrote the section on aging responses, organized different sections of the manuscript, and finalized the manuscript for submission.

## Conflict of Interest Statement

The authors declare that the research was conducted in the absence of any commercial or financial relationships that could be construed as a potential conflict of interest.
